# Gallstone Ileus as an Occult Cause of Small Bowel Obstruction and Subsequent Large Bowel Obstruction: A Report of a Rare Case

**DOI:** 10.7759/cureus.74912

**Published:** 2024-12-01

**Authors:** Matthew Luckman, Rebecca Ha, Alexander H Vu, Jane Han, Adam Golden, Jesse Victory

**Affiliations:** 1 Surgery, Georgetown University School of Medicine, Washington, USA; 2 Neurobiology, University of California San Diego, San Diego, USA; 3 General Surgery, New York University (NYU) Langone Health, New York City, USA

**Keywords:** acute large bowel obstruction, acute small bowel obstruction, gallstone diseases, gallstone ileus, rigler's sign

## Abstract

Gallstone ileus, a rare cause of mechanical bowel obstruction, occurs due to the formation of a cholecystenteric fistula allowing gallstones to migrate into the gastrointestinal tract. The condition occurs mostly in elderly patients, particularly women, and carries a significant mortality risk due to delayed diagnosis. This case report discusses a 77-year-old female patient with a history of chronic medical conditions, who self-presented with periumbilical pain, nausea, and reduced bowel movements. Initial imaging revealed pneumobilia and small bowel obstruction, leading to a diagnosis of partial obstruction attributed to adhesions. Despite surgical intervention and temporary symptom relief, the patient's condition deteriorated due to a subsequent colonic obstruction.

The case was complicated by delayed recognition of gallstone ileus, as imaging initially misinterpreted the obstructive mass as a "stool ball" rather than a gallstone. Following diagnostic laparoscopy and subsequent exploratory surgeries, the patient was found to have a gallstone impacted in the rectum, leading to colonic ischemia and perforation. This resulted in progressive renal failure, respiratory failure, and ultimately, the patient's death in hospice care.

This case underscores the diagnostic challenges of gallstone ileus and highlights two key delays: misattribution of obstructive symptoms to adhesions and failure to recognize colonic obstruction due to gallstone ileus. Early use of contrast-enhanced imaging and a high index of suspicion are crucial for timely diagnosis. This case emphasizes the importance of thorough inspection of the small bowel and ileocecal region during laparoscopy and the need for careful evaluation of imaging findings to improve patient outcomes in gallstone ileus cases.

## Introduction

Pathophysiology

Gallstone ileus is a rare form of mechanical bowel obstruction and is a primary complication of cholelithiasis which occurs in 0.3-0.5% of all cases [1,2]. Gallstone ileus results from gallstones forming inflammatory adhesions or pressure necrosis between the gallbladder and surrounding gastrointestinal (GI) structures. This communication can result in erosion and subsequent formation of cholecystenteric fistula, which allows gallstones to move freely into the GI tract [3]. This leads to the classic “rolling” obstruction, which presents as intermittent abdominal pain, nausea, and vomiting. Enteric gallstones commonly obstruct the terminal ileum [4,5].

Epidemiology

Gallstone ileus has been found to have a higher prevalence among the elderly, accounting for 25% of all cases of non-strangulating mechanical small bowel obstruction [5-7]. It is argued that this drives the reported 6.7% to 22.7% mortality rate [5,7-11]. Additionally, 70% of gallstone ileus cases have been observed in women [12,13].

Diagnosis

Symptoms and signs of gallstone ileus are often nonspecific and patients may have distension, nausea, vomiting, constipation, and intermittent abdominal pain [1,14]. A literature review shows that diagnostic imaging is a point of contention because of the subjectivity among observers and the inconsistent presentation of gallstones. The classic radiographic criteria of gallstone ileus are Rigler’s triad (also called Rigler’s sign): pneumobilia, intestinal obstruction, and ectopic gallstone [8]. Though abdominal radiography is vital in the disease’s management, only 15% of cases show all three components due to the radiolucent gallstones [15]. Early oral and/or intravenous (IV) contrast-enhanced computed tomography (CT) scanning is often considered the “gold standard” for diagnoses of intra-abdominal abnormalities such as gallstone ileus. Contrast-enhanced CT for gallstone ileus is cited with a 90-93% sensitivity, 100% specificity, and 99% accuracy [12].

For both contrast and noncontrast CT, however, the identification of gallstones varies highly based on the composition and structure of the gallstone. Gallstone identification is highly dependent on reasonable suspicion and skill of the viewer, which complicates timely diagnosis [16].

Management of gallstone ileus with small bowel obstruction and subsequent large bowel obstruction

This case is of interest due to the biphasic symptomatic presentation of gallstone ileus with small bowel obstruction and subsequent large bowel obstruction with necrotic perforation and pneumoperitoneum leading to acute respiratory failure and death.

Our comprehensive literature review revealed no previously described cases of mortality following colonic necrosis from complications due to gallstone ileus (Appendix 1). For the literature review of gallstone ileus management, an additional search strategy was deployed and delineated in Appendix 2. 

This case report has been reported in line with the SCARE Criteria. 

## Case presentation

A 77-year-old woman self-presented to the emergency department with periumbilical pain for five days. She had a medical and surgical history of atrial fibrillation, complete heart block status post pacemaker implant, type II diabetes mellitus, hypothyroidism, myelodysplastic anemia, normal colonoscopy two years prior, and endometrial carcinoma for which she received a hysterectomy in 2004 and has since been in remission. She also reported a history of chronic diarrhea since receiving radiation therapy for her cancer with an uncharacteristic decrease in bowel movements associated with emesis and bloating two days before presentation.

**Figure 1:**
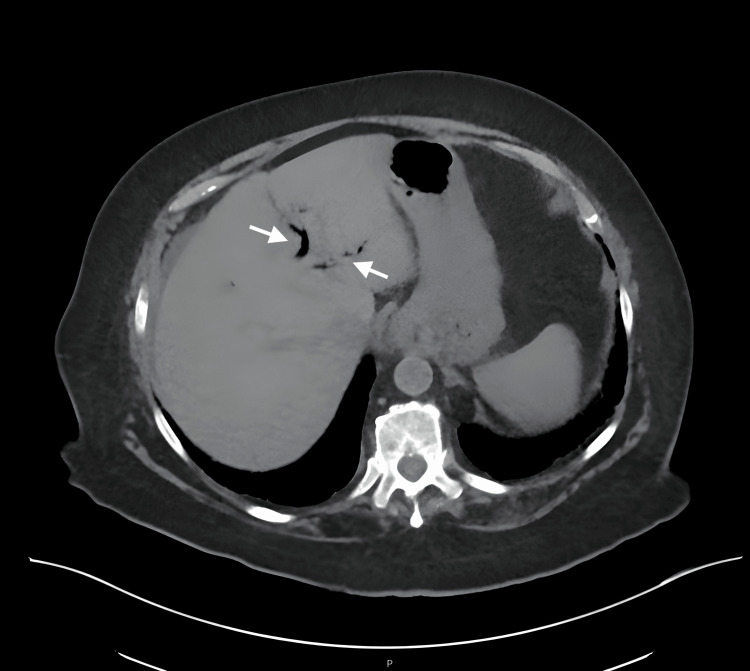
Anteroposterior (AP) CT imaging in the axial plane demonstrating subtle air within the biliary tree (arrows)

A physical exam revealed normal vital signs and a soft, minimally distended abdomen with minimal tenderness in the lower quadrants. Initial admission labs included a white blood cell count of 13x10^3/uL (reference range: 4.5 - 11.0 x 10^3/uL), blood urea nitrogen of 89 mg/dL (reference range: 7 - 18 mg/dL), creatinine of 2.25 mg/dL (reference range: 0.6 - 1.2 mg/dL), and lactic acid of 1.4 mmol/L (reference range: 0.5 - 2.2 mmol/L). CT of the abdomen and pelvis without contrast was obtained by the Emergency Department due to concern for acute kidney injury and revealed a small amount of pneumobilia and “mildly dilated small bowel to the level of the distal ileum where there is abrupt caliber change reflecting a transition point” (reported in the summary of the radiology read) (Figure 1). Given her mild presentation, she was diagnosed with partial small bowel obstruction and admitted for fluid resuscitation while nil per os (NPO).

**Figure 2:**
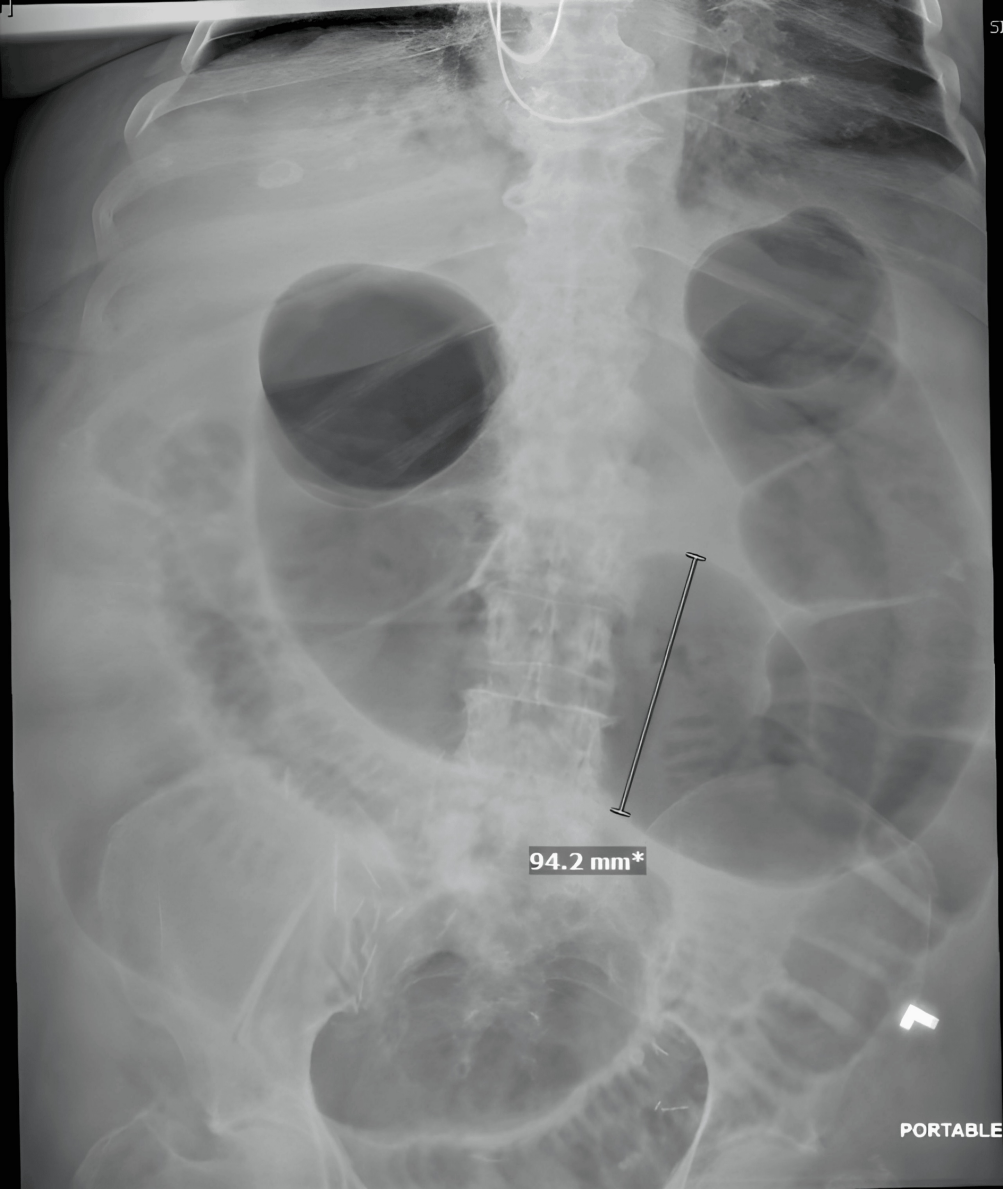
Abdominal film demonstrating large bowel obstruction after initial surgery.

**Figure 3:**
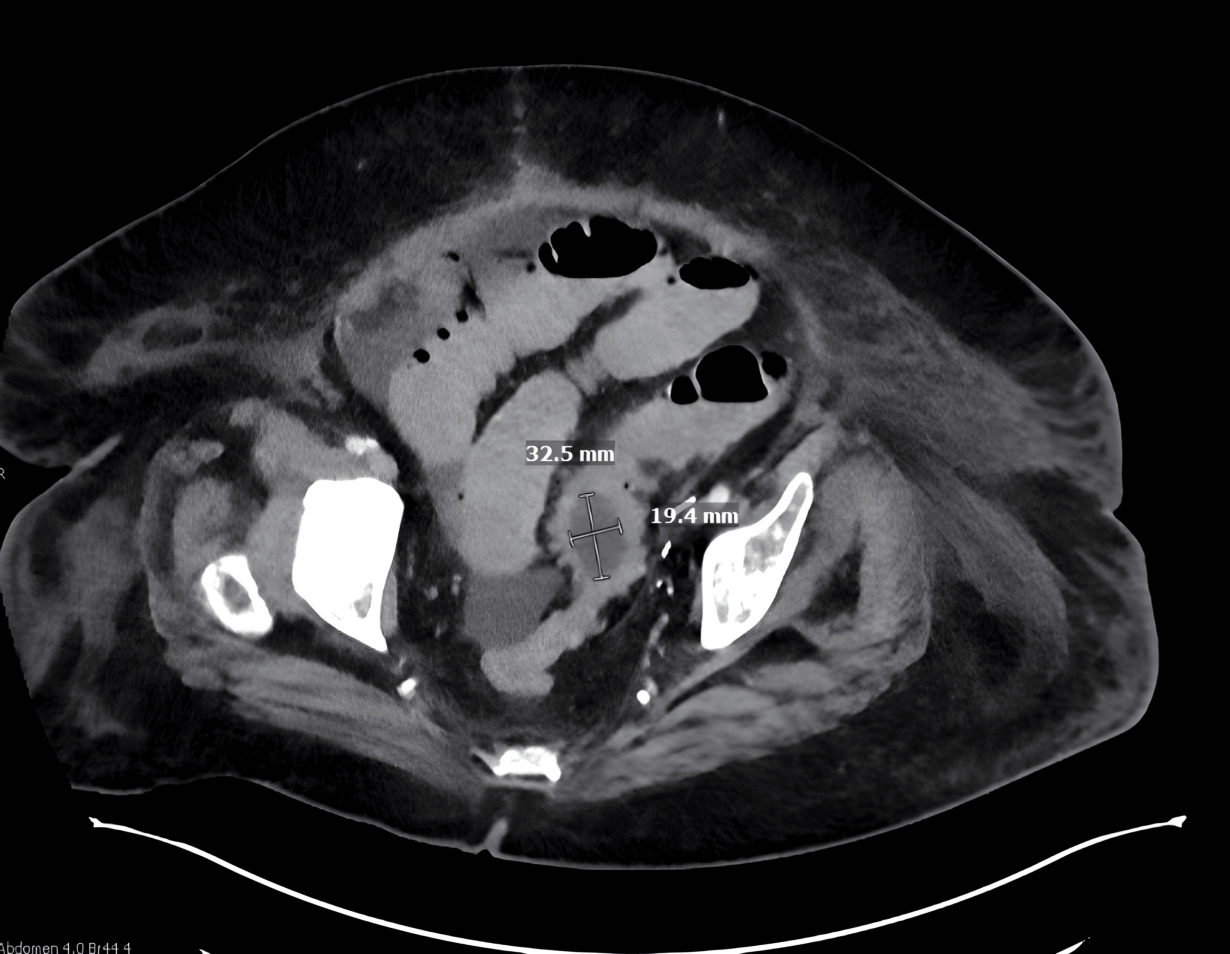
Abdominal CT gallstone mistaken for fecalith/stool.

The following day, her symptoms and exam worsened; therefore, a nasogastric tube was placed for decompression, and a subsequent Gastrografin challenge with water-soluble enteric contrast was performed. Given the lack of contrast progression into the colon, she was diagnosed with small bowel obstruction and taken to the operating room for diagnostic laparoscopy the following morning. An adhesive band was found extending from the cecum to the abdominal wall, which was believed to be the likely source of the obstruction because the terminal ileum was kinked around this band. Once the band was lysed, the change in small bowel caliber started to equalize immediately. Omental adhesions were found along the lower midline abdominal wall, but these were not associated with the bowel, which was dilated but otherwise healthy and free of adhesions. Postoperatively, she had a return of bowel function in the form of diarrhea and started tolerating oral intake. However, five days later, her abdomen was distended again, and her white blood cell count increased from 13.8 to 24.4x103/uL. Abdominal X-ray showed that the distention was mostly colonic, the patient was without pain or tenderness and still passing small amounts of diarrhea, so the diagnosis was presumed to be colonic ileus with the possibility of Clostridioides difficile infection (stool culture reported negative two days later) (Figure 2). Repeat CT of the abdomen and pelvis with IV contrast revealed a diffusely dilated large bowel to the level of the sigmoid colon. Radiology also reported “A 3.3 cm radiolucent structure within the lumen of the sigmoid colon just proximal to the change in caliber likely represents a stool ball (Figure 3). Short segment pneumatosis within the posterior wall of the cecum has the imaging appearance of benign pneumatosis.”

**Figure 4:**
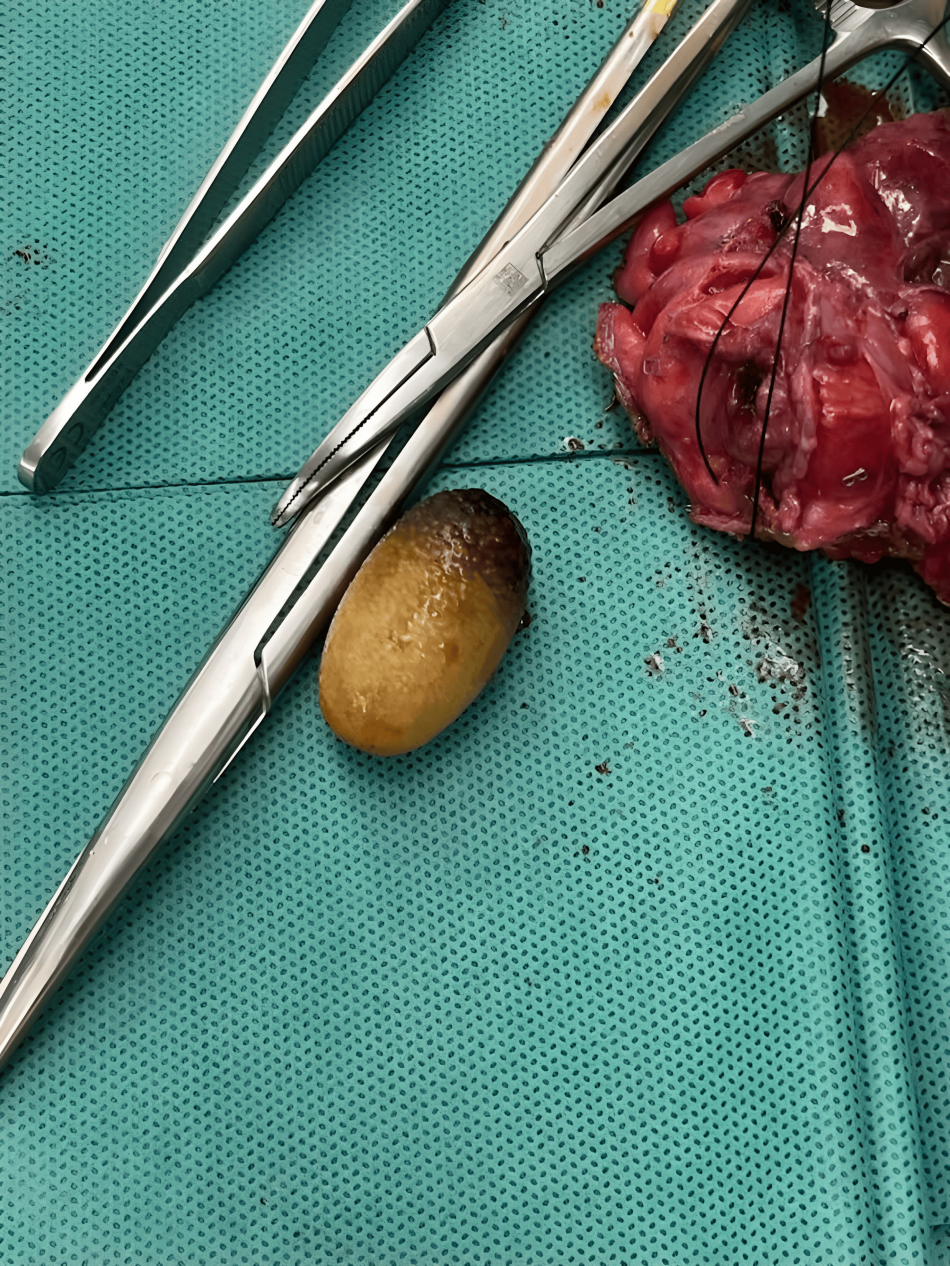
Large gallstone obstructing the rectosigmoid after subtotal colectomy.

Lactic acid was found to be 4.2 increasing to 5.0 mmol/L despite fluid resuscitation, so the patient was taken back to the operating room for an exploratory laparotomy. The entire colon was found to be ischemic, and there was feculent peritonitis originating from a perforation in the transverse colon. A damage-control resection was performed, as well as a subtotal colectomy. The patient was transferred to the surgical intensive care unit for resuscitation and returned the following day for a subsequent operation to remove the rest of the distal sigmoid colon and proximal rectum which revealed that the “stool ball” reported on the CT scan was a gallstone impacted in a strictured rectum (Figure 4). Two additional re-explorations over 48 hours led to a distal 15 cm small bowel resection and eventual ileostomy and final closure.

Over the following two weeks, the patient’s intensive care unit course was complicated by progressive renal failure and acute respiratory failure, which required continuous renal replacement therapy and tracheostomy. The patient was initially stabilized but then acutely decompensated again with findings of pneumoperitoneum consistent with another bowel perforation. A decision was made by the surrogate decision maker to transition to hospice, in which the patient expired.

Differential diagnosis for initial pneumobilia includes the following: (i) Gallstone ileus; (ii) Biliary tract procedure (typically resolved within months with competent sphincter of Oddi); (iii) Cholangitis; (iv) Emphysematous cholecystitis; (v) Trauma.

## Discussion

Due to the nonspecific presentation of gallstone ileus, diagnosis remains difficult and often results in delays. In many cases, the gallstone lodges in the narrowed ileocecal junction which causes the small bowel obstruction. This establishes the characteristic Rigler's triad (pneumobilia, ectopic gallstone, and small bowel obstruction) on plain film radiographs. Given the high mortality burden due to either delayed or misdiagnosis, this warrants a diligent review of abdominal radiographs and cross-sectional imaging in patients with preceding signs of biliary colic followed by obstructive symptoms. The case reported here highlights a missed gallstone ileus diagnosis, developing a colonic obstruction, progressive renal failure and acute respiratory failure, and death in hospice care. We identified two key errors that delayed the diagnosis of gallstone ileus, resulting in eventual death.

The initial error involved failing to recognize the signs indicative of Rigler’s triad, instead attributing the obstructive findings to adhesive bands from the patient’s previous surgical hysterectomy. These bands may have been the location of the initial obstruction by the gallstone, which eventually continued onward, possibly due to the laparoscopic exploration and manipulation. In the first CT scan, pneumobilia was noted along with a small bowel obstruction, representing the first two signs of Rigler’s triad. The initial radiographs could not appreciate the initial gallstone, which was made more difficult by the lack of IV contrast; however, a subsequent CT scan revealed a 3.3 cm hypodense oval mass within the lumen of the sigmoid colon. This mass was misinterpreted as a “stool ball,” later identified as a gallstone after its removal (completing the third component of Rigler’s triad). Traditionally, abdominal CT images depict stool as partially formed, heterogeneous, and intermediate-density within the colonic lumen [17]. Though only two components of Rigler’s triad were obtained before the initial diagnostic laparoscopy, scanning for causes of mechanical obstruction such as gallstones may be advantageous during the procedure.

Subsequent abdominal imaging could not identify the original cholecystoduodenal fistula caused by the gallstone as it passed from the gallbladder to the duodenum. Autopsy findings and findings at re-operations have shown that when persistent cholelithiasis is absent, biliary-enteric fistulas will close spontaneously once a distal obstruction is relieved [5,7]. Given the patient's prior hysterectomy, the small bowel obstruction was thought to be caused by adhesive bands, which were consequently lysed. Upon removal of adhesive bands, the patient’s bowel function improved, and they started tolerating oral nutrition, leading to the continued belief that gallstone ileus was not the cause of the symptoms. During the initial diagnostic laparoscopy, it was believed that the gallstone passed through the ileocecal valve and into the colon, subsequently resolving the obstructive symptoms. 

The second error was the delay in recognizing the etiology of the subsequent colonic obstruction, which presented as diarrhea and large bowel distention. These symptoms were initially thought to be attributed to Clostridium difficile and cultures were sent out to be tested but came back negative. Some strains of Clostridium difficile are toxin-producing which can lead to toxic megacolon, which clinically presents with diarrhea and >5.5cm distention of the large bowel on plain radiograph films [18,19]. The process resulted in a continued delay in diagnosis, which may have contributed to the development of ischemic colon and perforation.

Early and accurate imaging, combined with a high index of suspicion, is crucial for timely intervention of gallstone ileus. Though it may not be clinically indicated, if pneumobilia is detected on a radiograph, gallstone ileus could be considered as a possible differential diagnosis. We recommend thoroughly inspecting the small bowel and ileocecal valve during an indicated diagnostic laparoscopy to exclude gallstone ileus as a cause. The findings from this case, particularly the unusual radiographic characteristics of the gallstone, may aid in recognizing similar presentations and improving outcomes for future patients. This case exemplifies the complexities and severe potential outcomes of gallstone ileus, reinforcing the need for vigilance and prompt, effective management strategies in patients presenting with compatible symptoms.

## Conclusions

This case report underscores the importance of early and accurate diagnosis in managing gallstone ileus, a rare but potentially life-threatening complication of cholelithiasis. The delayed recognition of gallstone ileus in our patient led to severe complications, including colonic ischemia, perforation, and death. The delay in diagnosis was primarily due to the misidentification of Rigler’s triad on the CT scan and the failure to recognize the etiology of the subsequent colonic obstruction, which was initially thought to be attributed to Clostridium difficile. This case highlights the necessity of a high index of suspicion and thorough imaging evaluation in patients presenting with non-specific gastrointestinal symptoms. Prompt and accurate identification of gallstone ileus can significantly improve patient outcomes, emphasizing the need for thorough small bowel/ileocecal valve inspection during diagnostic laparoscopy and identification of signs of Rigler’s triad during diagnostic imaging.

## Appendices

Appendix 1: PubMed search strategy for cases of mortality following colonic necrosis

("colon"[MeSH Terms] OR "colon"[All Fields] OR "colonic"[All Fields] OR "colons"[All Fields] OR "colon s"[All Fields] OR "colonal"[All Fields] OR "colonically"[All Fields] OR "colonitis"[All Fields]) AND ("necrose"[All Fields] OR "necrosed"[All Fields] OR "necrosi"[All Fields] OR "necrosing"[All Fields] OR "necrosis"[MeSH Terms] OR "necrosis"[All Fields] OR "necroses"[All Fields]) AND (("gallstones"[MeSH Terms] OR "gallstones"[All Fields] OR "gallstone"[All Fields]) AND ("ileus"[MeSH Terms] OR "ileus"[All Fields]))

Appendix 2: PubMed search strategy for literature review of gallstone ileus management

(gallstone ileus) AND ((large bowel bowel obstruction) OR (colonic) OR (sigmoid) OR (coleus) OR (rectosigmoid))

Filters applied: Clinical trial, meta-analysis, randomized controlled trial, review, systematic review, with a limited date range of 2010 to present; a total of 17 articles were obtained, and ultimately four articles were used for literature review after rigorous manual screening by all authors.  
